# Predicting Ecological Momentary Assessments in an App for Tinnitus by Learning From Each User's Stream With a Contextual Multi-Armed Bandit

**DOI:** 10.3389/fnins.2022.836834

**Published:** 2022-04-11

**Authors:** Saijal Shahania, Vishnu Unnikrishnan, Rüdiger Pryss, Robin Kraft, Johannes Schobel, Ronny Hannemann, Winny Schlee, Myra Spiliopoulou

**Affiliations:** ^1^Knowledge Management and Discovery Lab, Otto-von-Guericke University Magdeburg, Magdeburg, Germany; ^2^Institute of Clinical Epidemiology and Biometry, University of Würzburg, Würzburg, Germany; ^3^Institute of Databases and Information Systems, Ulm University, Ulm, Germany; ^4^DigiHealth Institute, Neu-Ulm University of Applied Sciences, Neu-Ulm, Germany; ^5^Sivantos GmbH - WS Audiology, Erlangen, Germany; ^6^Department of Psychiatry and Psychotherapy, University of Regensburg, Regensburg, Germany

**Keywords:** contextual multi-armed bandits, mHealth for tinnitus, EMA prediction, similarity-based prediction, prediction on sparse data, prediction on time series with gaps, prediction in mHealth data

## Abstract

Ecological Momentary Assessments (EMA) deliver insights on how patients perceive tinnitus at different times and how they are affected by it. Moving to the next level, an mHealth app can support users more directly by predicting a user's next EMA and recommending personalized services based on these predictions. In this study, we analyzed the data of 21 users who were exposed to an mHealth app with non-personalized recommendations, and we investigate ways of predicting the next vector of EMA answers. We studied the potential of entity-centric predictors that learn for each user separately and neighborhood-based predictors that learn for each user separately but take also similar users into account, and we compared them to a predictor that learns from all past EMA indiscriminately, without considering which user delivered which data, i.e., to a “global model.” Since users were exposed to two versions of the non-personalized recommendations app, we employed a Contextual Multi-Armed Bandit (CMAB), which chooses the best predictor for each user at each time point, taking each user's group into account. Our analysis showed that the combination of predictors into a CMAB achieves good performance throughout, since the global model was chosen at early time points and for users with few data, while the entity-centric, i.e., user-specific, predictors were used whenever the user had delivered enough data—the CMAB chose itself when the data were “enough.” This flexible setting delivered insights on how user behavior can be predicted for personalization, as well as insights on the specific mHealth data. Our main findings are that for EMA prediction the entity-centric predictors should be preferred over a user-insensitive global model and that the choice of EMA items should be further investigated because some items are answered more rarely than others. Albeit our CMAB-based prediction workflow is robust to differences in exposition and interaction intensity, experimentators that design studies with mHealth apps should be prepared to quantify and closely monitor differences in the intensity of user-app interaction, since users with many interactions may have a disproportionate influence on global models.

## 1. Introduction

According to De Ridder et al. (2021), “The capacity to measure the incidence, prevalence, and impact will help in identification of human, financial, and educational needs required to address acute tinnitus as a symptom but chronic tinnitus as a disorder.” Mobile health apps for tinnitus have the potential of assisting patients in self-assessment of their condition and of delivering insights on tinnitus heterogeneity to the medical experts, as reported by Probst et al. (2017); Cederroth et al. (2019), and Pryss et al. (2019, 2021) among others. This is particularly the case for mHealth apps that collect Ecological Momentary Assessments (EMA): several studies on mHealth tinnitus apps have demonstrated that EMA recordings deliver insights on tinnitus stages during the day and on the interplay of personal traits and severity of tinnitus symptoms (see, e.g., Probst et al., 2017; Mehdi et al., 2020; Unnikrishnan et al., 2020a; Jamaludeen et al., 2021).

Modern mHealth apps are able to deliver suggestions to the app users, exploiting knowledge about the users' prior behavior (Martínez-Pérez et al., 2014) or behavior change (Mao et al., 2020), and they contribute also to clinical decision support (Watson et al., 2019). In the context of tinnitus, TinnitusTipps^1^ delivers information toward “health literacy” and suggestions that promote well-being, e.g., suggestions on physical exercising. Personalization of such suggestions implies taking account of a user's personal traits and needs, and has the potential of improving user experience and of anticipating undesirable developments in the user's condition. However, personalization demands the ability to learn from past EMA and to predict future EMA.

In contrast to passive recordings, e.g., of ambient noise, EMA recordings demand action by the app user. Some studies, as by Probst et al. (2017), concentrate on users who have many recordings. While such studies are beneficial for the acquisition of insights on how tinnitus is experienced in general, they contribute less toward personalized services, which need to learn and predict for each user, even for users who interact rarely with the app and deliver too few data. Schleicher et al. (2020) model the intensity of the interaction with the app as adherence and attempt to identify adherence patterns. Other studies, as Unnikrishnan et al. (2020a) and (Prakash et al., 2021) and the earlier version of this work (Shahania et al., 2021), investigate ways of learning from users who deliver very little data. The main objective of these studies is to provide tools that predict future EMA of a user, thus forming a basis for the prediction of undesirable events and for the design of personalized services. The main challenge of such methods is the scarcity of data for some users.

Technically speaking, a method that learns from all the data of all the users induces a “global model.” It abstracts from the idiosyncracies in the EMA recordings of each user and attempts to predict the future condition of a user from all that is known on the past EMA of all users. In contrast, a method that learns from the data of a single user, builds a model peculiar to that user, capturing the user's idiosyncracies and exploiting them for future predictions. Such a method is bound to learn from as little data as are available for each user, but suffers from the obvious disadvantage that some users have too few EMA recordings for any reliable prediction of their future EMA. For such users, it seems reasonable to exploit data of other, similar users for predictive modeling. Indeed, in our earlier works (Unnikrishnan et al., 2020a, 2021), we have shown that methods which exploit similarity among users can predict future EMA recordings of users with little data. We term a model learned on the data of a user and users similar to him/her as a “local model,” to highlight the fact that such a model exploits only data in the user's vicinity (in a multi-dimensional space spanned over the static data of the users and their EMA recordings). A model learned on the data of the user without his/her neighbors is a special case of local model that considers only a single user.

There is a well-known separation of methods into idiographic ones that learn for each individual separately and nomothetic ones that aim to explain the common traits of all individuals. Without entering the conceptual debate of idiographic vs nomothetic approaches (cf. the elaboration of Hermans, 1988 among many), we point out that from the technical perspective, a nomothetic approach builds a global model, an idiographic approach builds a local model for each single individual, while an approach that exploits information on a user and the users similar to her is a nomothetic approach that builds a local model by concentrating only on few users that are similar to a given user. Since our methods use the same machine learning instruments independently on whether they build global or local models, we avoid the distinction between nomothecy and idiography hereafter.

In this study, we build upon our earlier works on learning local models from little data (Unnikrishnan et al., 2020a), on comparing methods that learn from all data of all individuals to methods that learn local models (Unnikrishnan et al., 2019) and on frameworks for such comparisons (Shahania et al., 2021). We present a complete framework for studying how methods designed for little data can contribute to predicting the condition of a user interacting with a mHealth for chronic tinnitus. In a nutshell, we want to predict the next vector of answers in the best possible way. We investigate the following research questions:

1. **RQ 1:** To what extent can methods that learn from little-data deliver good predictions?
  · *RQ 1a:* How to exploit little-data for predictions?
  · *RQ 1b:* How to orchestrate the invocation of methods building local models, so that they are only invoked when their prediction is good?
  · *RQ 1c:* How to measure the superiority of a little-data method over a method that learns from all the data?
2. **RQ 2:** What factors influence the superiority of local vs. global models?
  · *RQ 2a:* How do different configurations of little-data methods affect their performance?
  · *RQ 2b:* How do different behavioral phenotypes affect the performance of local vs global models?

We address these research questions on the data of a clinical study involving patients with chronic tinnitus: the participants used an mHealth app to record daily EMA, and received regular non-personalized tips on good practices for living with tinnitus.

To answer RQ 1, we consider for streams. Their purpose is to capture the different interaction behavior of the entities (here: mHealth app users), which causes the variability of the data stream per entity. Hereby, the global model exploits the data stream of all entities, whereas the local models only use data of one entity (entity-centric local model) or an entity and its nearest neighbors.

To ensure that we chose the best configuration depending on the varying data streams, we incorporate the global and local models into a contextual multiarmed bandit (CMAB). This bandit employs a strategy to select one model for each time point based on past rewards and additional meta information of the entity. For RQ 2, we derive an evaluation procedure along with the factors that are responsible for selecting each type of model for different data streams. These factors include the history length of the entities, their personal traits, their time of arrival in the system, and the temporal proximity of the predictions.

This article is organized as follows. Section 2 covers the materials used for our study. Section 3 describes the state of the art techniques present in stream learning and describes techniques for the creation of multi-armed bandits. We are using those for defining our proposed approach. This section also contains details about the need to define global and local models to tackle our problem. Sections 4.2 and 4.3 depicts the results on RQ 1 and RQ 2 followed up with the discussion of the insights, findings, insights, and future work in Section 5.

## 2. Materials

For our work, we have considered the data from a clinical study with the mHealth app TinnitusTipps, conducted in 2018/2019. The study was approved by the Ethical Review Board of the University Clinic Regensburg. The ethical approval number is 17-544-101. All study participants provided informed consent. For more information on the inclusion/exclusion criteria of the participants, please refer to Schlee et al. (2022).

The TinnitusTipps app (see text footnote 1) was developed by computer scientists, psychologists, and Sivantos GmbH—WS Audiology (a company specialized in hearing aids). As we detail in our earlier work (Shahania et al., 2021), TinnitusTipps uses tips to promote “health literacy,” and engaging features to stimulate user involvement.

The study participants were split into three groups that differed on their exposition to the functionalities of TinnitusTipps. The tips were not personalized, so they were always delivered at random, independently of the group of the user.

- *Group A:* The users of the mHealth app received the tips for the entire project duration of 4 months (*n* = 11).
- *Group B:* In the first 2 months, the users received no tips. They only got the normal “TrackYourTinnitus” function (see Kraft et al., 2020), i.e., they only answered questionnaires. In the second half of the project, in months 3 and 4, they received the tips (*n* = 10).
- *Group Y:* This group is more similar to Group A, in the sense that they received tips from the beginning (*n* = 13). The difference between groups A and Y is that the participants have been supervised by different persons who followed slightly different protocols. For this reason, we skipped group Y and concentrated on groups A and B that followed the same study protocol.

The study protocol encompasses two questionnaires, one to be filled at registration and one EMA questionnaire to be filled at least once a day. The registration questionnaire is the “Tinnitus Sample Case History Questionnaire” TSCHQ (Langguth et al., 2007), consisting of 31 items. The EMA questionnaire consists of the 8 items depicted on Table 1.

**Table 1:** Items S01 to S08 from the EMA questionnaire of TinnitusTipps—the range of values for the answers of the items S02-S07 is between 0 and 100 (first two columns shown also in Shahania et al., 2021).

**Item**	**Question description**	**Short description**
S01	Do you perceive the tinnitus right now?	–
S02	How loud is your tinnitus right now?	Tinnitus loudness
S03	How distressed are you by your tinnitus right now?	Tinnitus distress
S04	How well do you hear right now?	–
S05	How much are you limited by your hearing right now?	–
S06	How stressed do you feel right now?	stress
S07	How exhausted do you feel right now?	–
S08	Are you wearing a hearing aid right now?	–

*For S01 and S08, we have binary answer (yes/no)*.

We removed all users of group Y and those belonging to no group, i.e., we considered groups A and B only. An extensive exploratory analysis of the data of these users appears in the following section. It includes the distribution of the values of the answers to the EMA questionnaire items for each group, a correlation analysis among the EMA items independently of the groups, and a correlation analysis among the EMA items for each group.

Before addressing the research questions, we performed an exploratory analysis reported hereafter. For the RQs themselves, we skipped the categorical items S01 and S08. Due to space limitations, for some results on the RQs, we report only on the items S02, S03, S07 (cf. 3rd column of the Table 1). All results are in the Supplementary Material.

## 3. Our CMAB-Based Method

**Figure 1:**
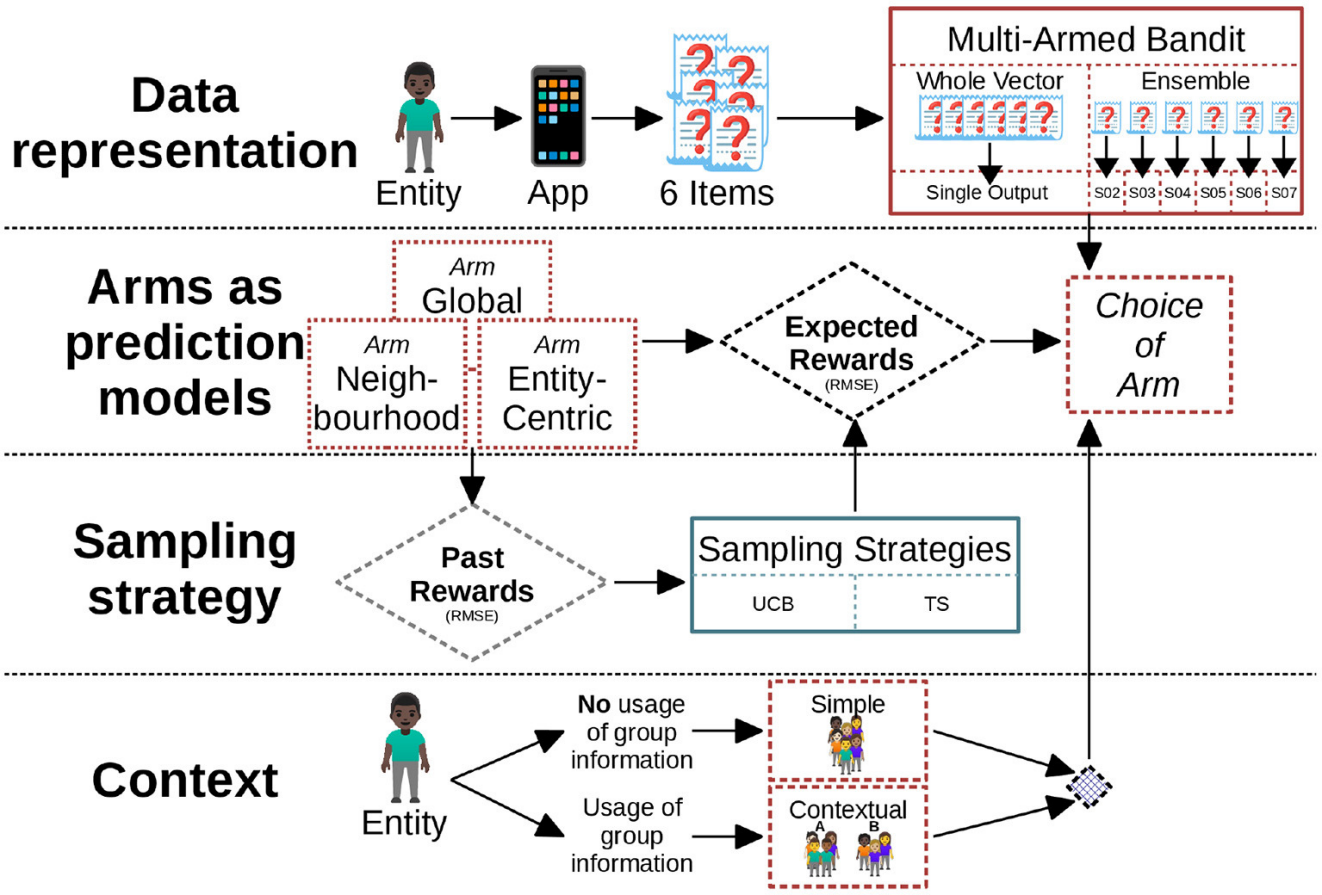
The figure shows the outline of the system used to predict the answers given by the entities. The entire architecture is divided into 4 main parts. The first part shows the representation of the data and the output. The second part is about the prediction models as the arms of the bandit followed by the sampling strategies and the context for the bandit. We have both ensemble and whole vector bandit for each contextual and simple bandits. RMSE here refers to root mean square error.

The architectural design for this article depends on several factors, which are depicted in Figure 1. At each time point, we want to predict the vector of answers. We have the large architecture to do so but in the end CMAB chooses the arm that is expected to be the best prediction. The expectation of quality of the prediction is quantified as reward. This aids us in deciding when local models are preferred over global models based on the behavior of this bandit (cf. RQ 1b). Therefore, the core factors of investigation are as follows:

1. Data representation : Depending on the structure and features of the data, the bandit has to be designed accordingly. User's answers to the items constitute a multidimensional vector of features. We distinguish between representations for an ensemble of bandits, where each ensemble member has a predictor for one of the items, and a whole vector bandit, where all the items are predicted as one vector by a single bandit.
2. Arms as prediction models : The bandits choose the arm to pull depending on the expected reward, informally the quality of the prediction. We consider three arms: one arm considers only the past data of the user for which the prediction must be made (entity-centric arm), one arm additionally considers the past data of the user's k nearest neighbors (neighborhood arm), and one arm considers the past data of all users (global arm). Each arm of the bandit can be handled in the same way or rules can be made to trigger the arms differently.
3. Sampling strategy: Since an arm is chosen based on the expected rewards, past rewards of when an arm was chosen are sampled in a heuristics called sampling strategy.
4. Context: If the bandit makes use of additional meta-information regarding the current sample we call it context. In our case, the context of observation is the group, to which the entity referred by this observation belongs. We consider one *contextual bandit* which considers as context the group (A or B) to which the user belongs, i.e., considers only data from users of this group when building the predictors (cf. point 2). We also consider *simple bandit* that has no context, i.e., does not take the groups into account.

In summary, we employed 4 different configurations which are based on the variations of the context and data representations. These configurations are as follows:

1. Contextual Whole Vector Bandit: CWB
2. Simple Whole Vector Bandit: SWB
3. Contextual Ensemble Bandit: CEB
4. Simple Ensemble Bandit: SEB.

The SEB configuration means that our bandit does not use any additional information to decide which arm to use but instead completely relies on the sampling strategy being employed but for each item, there is a different bandit provided. Thus, the SEB indirectly has some contextual knowledge about the items but none about the complete sample. On the other hand, CWB configuration uses meta-information about the sample for its decision but provides only a single output for all the items and due that has no contextual knowledge about them. Furthermore, the SWB configuration is a single bandit that uses no contextual information about either the samples or the items, whereas the CEB is provided with the context of both things.

### 3.1. Data Representation: Entities and Their Observations

The data are comprised of two main components: the entities and their stream of observations. Hereby every entity is a user of the system, which is represented by observations in the form of 6-dimensional multivariate time series (cf. items of the EMA questionnaire in Table 1). The time series of the user is the sequence of calendar times when we have seen the user. *t*_0_ is the first time and *t*_*n*_ is the last time when we see the user. Let u be a user, and *t*_*u*,0_, *t*_*u*,1_, …, *t*_*u*,_*n*__*u*__ be the time steps at which the user was observed. These are calendar time steps, i.e., they contain a date and clock as hh:mm:ss. An entity might interact with the app in different intervals, therefore each entity contains a different amount of observations and the time between observations might be hours to days. Therefore an observation *o*_*i*_ at a time steps *t*_*i*_ is an element of the time series for a particular entity and might contain null (NaN) values. For us a time point *t*_*i*_ is not a specific point of date, but instead an answer is given from any entity, i.e., we sort the observations by their date of appearance and label the sorted list as time steps. Therefore there is no missing time step. But since the time step *t*_*i*_ is dependent on the entity's interaction with the mHealth app, the difference between time steps *t*_*i*−1_ and *t*_*i*_ can range between a day to a week. Furthermore, at a particular *t*_*i*_, if there is a NULL value corresponding to some questions, we replace it with the value from the prior time step *t*_*i*−1_.

As discussed in point 1 the output of the bandit has two major options namely whole vector and ensemble bandit. The whole vector bandit means that we provide a single output, i.e., a 6-dimensional vector, for all the items of the observation. In the ensemble setting, we instead use one bandit per item that returns a scalar output. It would be possible to combine both approaches, i.e., combine certain items for one bandit but not all. We did not investigate this option for this article.

### 3.2. Arms as Prediction Models

As mentioned earlier, we predict the upcoming observations using a bandit. For this purpose, we have to decide the definition of the arms of the bandit which in our case are models used for prediction. To better support the different types of entities and their behavior including the number of observations per entity and thus exploiting also little data for our predictions (cf. RQ 1a), several choices are made:

1. The arm setup based on the entity's past observations (history) [RQ 1a],
2. the learning algorithm for the predictor [RQ 1a],
3. the performance measure for the arm [RQ 1b],
4. and the reward function for the bandit [RQ 1c].

#### 3.2.1. Arm Setup Based on the Entity's History (for (RQ 1a)

We design the bandit with 3 arms and hence have 3 different setups in place for prediction based on the number of entities and their respective observations. The settings are as follows:

1. *The global arm*: This is modeled based on the history of all the entities.
2. *The entity-centric arm*: This is modeled separately for each entity based on a history of only the entity itself.
3. *The neighborhood arm*: It is designed based on the history of the entity itself and its k nearest neighbors.

With the above settings, it is possible to focus on different properties of the data. On the one hand, the entity-centric arm is potentially better in exploiting the personal traits of an entity but information may not be always recent as there might be time gaps between the observations from a particular entity (cf. Section 3.1). On the other hand, the global arm can capture this temporal proximity but may over-generalize in predicting certain observations of entities. Hence, we introduce additionally the neighborhood arm which acts as a trade-off between the global and the entity-centric arms, capturing both the temporal proximity and the local behavioral patterns. Since both entity-centric and neighborhood arms capture the local behavioral patterns, we refer to them as local arms.

Based on how the arms are designed, all of them need a different number of observations before they can be used, i.e., the global arm needs only a fixed number of observations *m*, whereas the entity-centric arm requires *n* observations from the respective user. Therefore, the neighborhood arm additionally needs also observations from all its nearest neighbors, i.e., *k* · *n*. This implies that at the beginning not all arms would be equally available for the bandit, especially the neighborhood arm. We will keep the arm setting fixed in a rule-based manner. This means that arms can only be used when enough observations have been processed for the model to start predicting. Hence, the global arm is used as a fallback option when there are not enough observations available for the local arms. For our experiments, we need to set the respective number of observations *m* and *n*. Due to the global arm needing to generalize compared to local arms *m* should be sufficiently larger than *n*. Additionally, *n* needs to be smaller than the minimum history length in the data which is 12 in our case for entity 25. Based on these constraints, we chose *m* and *n* as 30 and 5, respectively, from prior experimentation. Similarly, *k* is set to 4 as it provided the best results for almost all the items based on preliminary analysis.

#### 3.2.2. Choice of a Learning Algorithm for the Predictor (for RQ 1a)

As discussed in the Section 3.1, the data constitute a stream of observations, so we considered following regression algorithms for streams:

1. Hoeffding Tree (Hulten et al., 2001)
2. Hoeffding Adaptive Tree Regressor
3. Adaptive Random Forest Regressor (Gomes et al., 2017).

The only difference between the normal Hoeffding Tree and the adaptive version is that for drift detection it uses ADWIN (Bifet and Gavaldá, 2007), which is an adaptive sliding window algorithm. In the preliminary experiments all the three algorithms performed mostly at par with each other but we decided to make use of the drift detection of the adaptive version. It is assumed that this will allow us to capture the drifts in data of the users, if any, in the future. The detailed descriptions of the algorithms can be found in the Supplementary Material.

#### 3.2.3. Performance Measure for the Arm (for RQ 1b)

The algorithms explored for prediction in Section 3.2.2 are regression models, therefore, the squared error is picked as the evaluation criterion. Since in other related stream classification papers (Unnikrishnan et al., 2020a) the root mean squared error (RMSE as in Equation 1) has been employed, we decided to go forward with the same.


(1) $$\begin{array}{l} \text{RMSE}=\sqrt{\frac{1}{N}\underset{i}{\sum }{\left(x_{i}-\hat{x_{i}}\right)}^{2}} \end{array}$$

For every target output and every time-step, RMSE value is calculated, where *x*_*i*_ is the actual value and $\hat{x_{i}}$ is the predicted value, while *N* is the number of observations. The RMSE value is averaged over all target outputs to understand the overall performance per time step.

#### 3.2.4. Choice of Reward Function for the Bandit (for RQ 1c)

Most of this articles in the related work used 1 − error from the models as their reward. At each time step *t*_*i*_, the bandit decides which arm to use for prediction based on past rewards, where the reward is the RMSE over all the answers to the items (cf. Equation 1), normalized on the upper bound of the error *U*, defined as the largest value minus the smallest value that each target can acquire. For our application scenario, *U* = 100 − 0 = 100, since all items we chose for our analysis have a value range from 0 to 100 (cf. Table 1).

### 3.3. Choice of Sampling Strategies for the Bandit

After the arms have been defined for the bandit, we need to decide the heuristic to choose an arm for the prediction of an observation. This choice is dependent on the sampling algorithms employed by the bandit. The inspiration for the sampling algorithms was taken from the related work explored. To compare various strategies Upper Confidence Bound (UCB) and Thompson Sampling (TS) were used to conduct the experiments. UCB is based on a simple principle that uncertainty regarding the arm is directly proportional to the importance of the exploration of an arm. So if the arm is very uncertain, UCB chooses this and picks the corresponding reward to this arm and makes the arm less uncertain. This goes on until the uncertainty is below some decided threshold. For our experimentation UCB1 was used which is formulated mathematically as follows:


(2) $$\begin{array}{l} Q\left(a\right)+\sqrt{\frac{2log\left(t\right)}{N_{t}\left(a\right)}} \end{array}$$

where *Q*(*a*) is the average reward of arm *a* for each round, *t* is the total number of rounds “played” thus far and *N*_*t*_(*a*) is the number of times arm *a* was selected thus far.

For each round/iteration/time-step, we play the arm that maximizes Equation (2). The first term in the Equation (2) controls exploitation, i.e., choosing the arm where the average is the largest, whereas the second term controls the exploration, hence trying to maintain the balance between both.

TS on the other hand makes use of modeling the rewards as probability distributions to sample the expected reward of an arm instead of using the average expectation like UCB does (Equation 2). Hence, the exploration is handled *via* the sampling process instead of an explicit representation in the formula. The advantage over UCB according to the survey is that already good arms are more likely to be exploited without forcing exploration based on uncertainty. Generally, this allows TS to converge faster to an optimal solution meaning the one that always chooses the model with the maximum reward. But this highly depends on the correct modeling of the reward. For our analysis, we considered normal distribution for sampling.

Additionally, to evaluate the applied strategies we have also considered the following sampling methods:

1. optimal: chooses an arm that outputs the maximum reward out of all;
2. worst: chooses an arm that outputs the minimum reward out of all;
3. random: chooses any arm randomly.

With the sampling strategies, we decided on the last building block for the bandit so that it can invoke the different arms when they are potentially most beneficial (cf. RQ 1b). This of course also is dependent on the arm itself and the reward function we have chosen for its representation.

## 4. Results

### 4.1. Exploratory Data Analysis

To acquire better insights on the data distribution of the EMA of group A and group B participants, we first performed a univariate analysis on the user's answer to each EMA item and then we computed the correlation matrix for the EMA items, using heatmap as a basis for each group. Also, a comprehensive analysis on the item S01 has been done to understand the role of perceiving tinnitus to the answers given by the user.

#### 4.1.1. S01 Analysis

To understand the differences between the tinnitus and non-tinnitus perception times of the user, the values of item S01 were analyzed for both the groups (cf. Table 2). In principle, Group B is more active than Group A. Independently of the group, we have fewer observations from the users in the next 2 months of the study. Users 17, 28 from Group A and users 29, 42 from Group B are the ones who do not perceive tinnitus most of the time. Group B users mostly said so in the first 2 months while the Group A users said so in both the first and the next 2 months of their study.

**Table 2:** Number of times users answer zero for S01 in relation to their total number of observations overall in their first 2 months and next 2 months of the study for group A and group B.

**Group A**					**Group B**				
	**First 2 Months**		**Next 2 Months**			**First 2 Months**		**Next 2 Months**	
**User**	**Total Observations**	**Perc. of 0s**	**Total Observations**	**Perc. of 0s**	**User**	**Total Observations**	**Perc. of 0s**	**Total Observations**	**Perc. of 0s**
17	108	69.4%	62	74.2%	20	132	0.0%	84	0.0%
18	50	22.0%	35	0.0%	24	102	0.0%	80	0.0%
19	20	20.0%	0	0.0%	25	11	72.7%	1	100%
22	157	0.0%	130	0.0%	29	169	34.9%	149	1.3%
23	129	0.8%	20	0.0%	35	190	0.0%	134	0.0%
28	150	49.3%	125	48.8%	42	58	34.5%	21	4.8%
30	101	0.0%	27	0.0%	47	165	1.2%	135	0.0%
31	104	18.3%	99	1.0%	48	173	0.6%	153	0.0%
33	39	12.8%	0	0.0%	51	259	0.4%	168	0.6%
40	24	16.7%	8	0.0%	52	45	26.7%	9	11.1%
43	50	0.0%	18	0.0%	-	-	-	-	-

#### 4.1.2. Univariate Analysis

For the univariate analysis, the box plots and distributions for each of the items are used to see how the overall spread is for the answers between 0 and 100. We observe that for all the variables except S04 for both the groups, the likelihood of having the value 0 is high. For variable S04, the values usually are between 20 and 100. For Group A the mode of S04 is around 80 and for Group B we have a trimodal distribution with modes around 50, 80, and 100. For S02 the largest mode is around 80 for both the groups but for Group A most of the values lie around 40. For S03 we have multi-modal distribution with a mode at around 30 and 85 for Groups A and B, respectively. For the items S05, S06, and S07, there is a strong shift toward the value 0 in Group B. On the contrary for Group A, the values lie on an average between 20 and 40, but also have a mode at 0. Furthermore, we inspected the value distributions of the individual users for each item and found that each user has their own “preferred” range of values. This strengthens our expectation that local models (idiographic ones and those based on neighborhood) will be predictive for some users.

#### 4.1.3. Bi-variate Analysis

**Figure 2:**
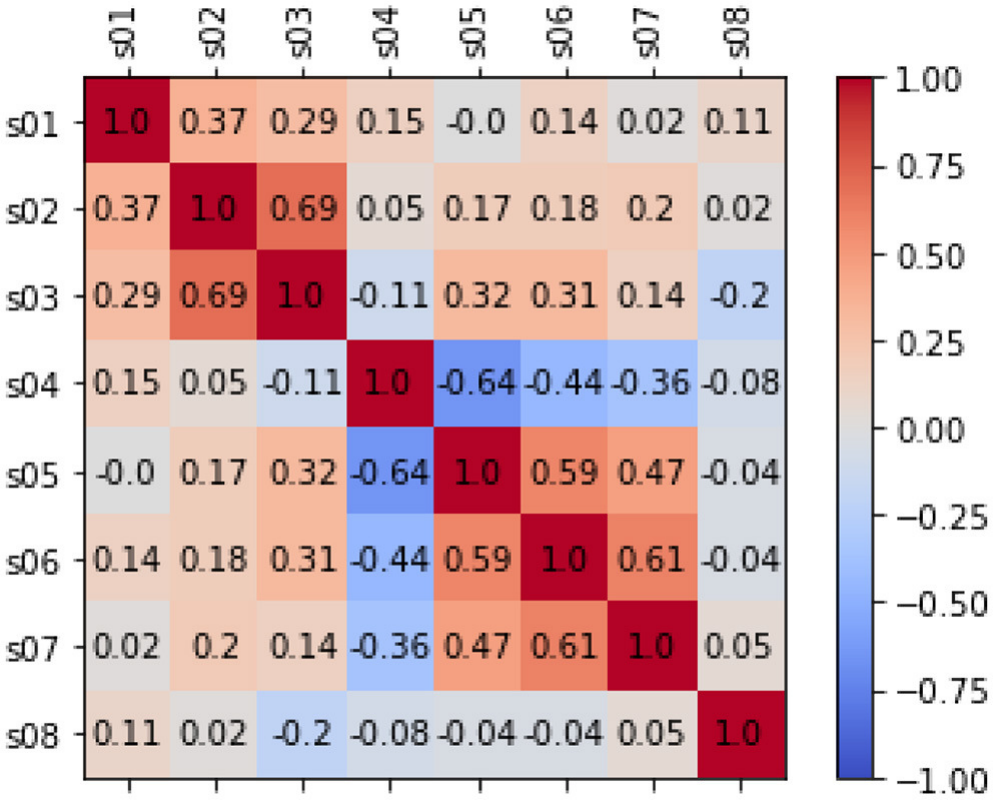
The figure shows correlation analysis on the EMA items based on Pearson's correlation. When there is no correlation between 2 variables (when a correlation is 0 or near 0) the color is gray. The darkest red means there is a perfect positive correlation, while the darkest blue means there is a perfect negative correlation. Additionally, the numbers inside each cell represents the absolute value of the correlation.

For the bivariate analysis among the EMA items, we put the observations of both groups together and temporarily ignored that some users contributed more observations than others. The heatmap of the analysis is depicted in Figure 2. We found that (independently of the users), tinnitus loudness (S02), and tinnitus distress (S03) are positively correlated and so are stress (S06) and exhaustion (S07). The item S04 on hearing is particular in that higher values are better: accordingly, it stands in negative correlation to stress (S06), exhaustion (S07), and limitations because of hearing (S05). There is a significant correlation between all the above correlations based on the p values before the Bonferroni correction.

### 4.2. Experiments for RQ 1

**Figure 3:**
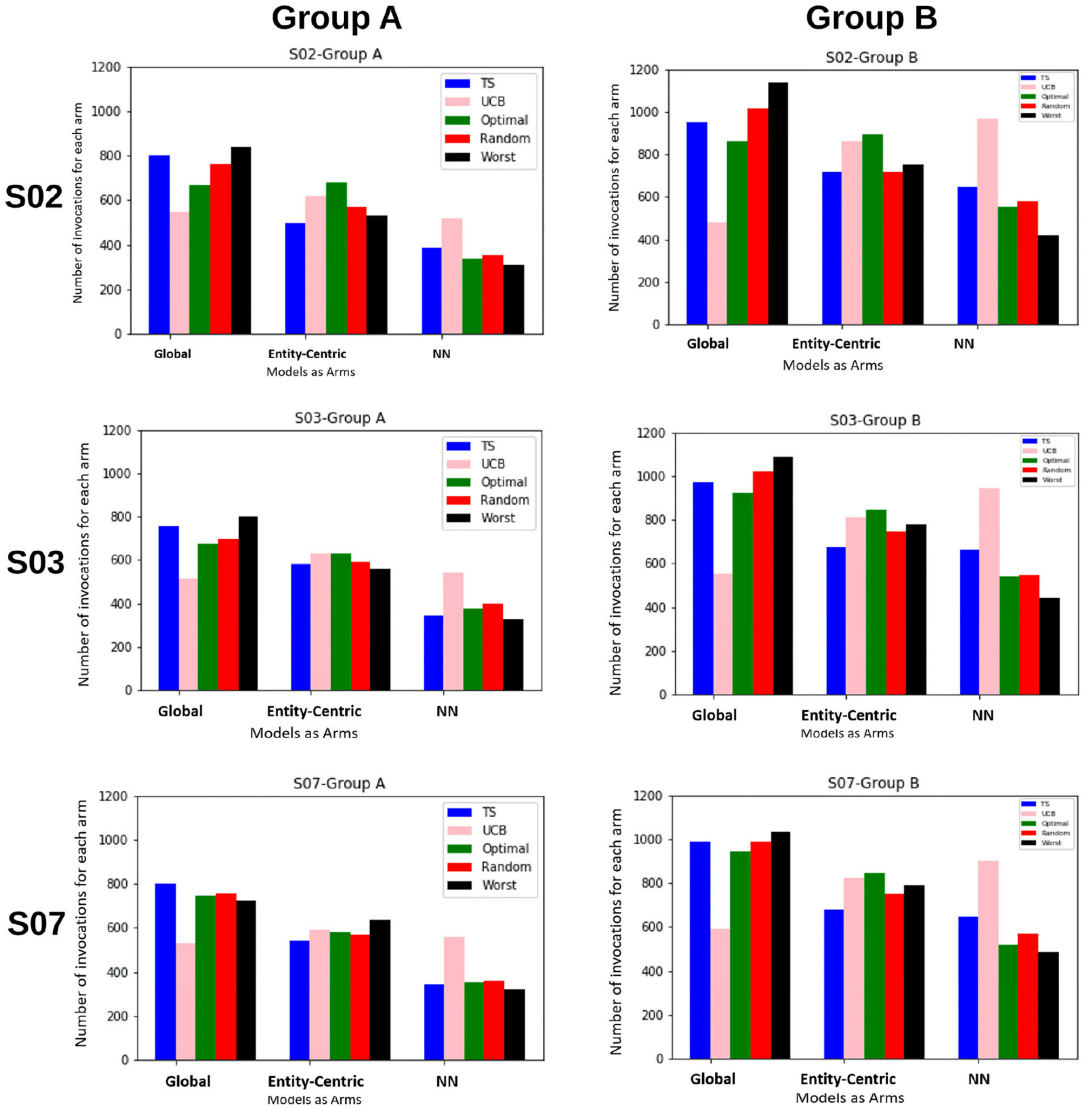
The figure shows for the ensemble bandit, the number of times each arm is invoked for the items by different samplers for both the groups. Five samplers as discussed in Section 3.3 above have been compared here.

In the first set of experiments, we are trying to evaluate if the methods taking into account the local behavioral patterns are superior to the global method. For this purpose, we are looking at how often the global, entity-centric, and neighborhood arms (NN) are chosen, therefore, their percentage of invocations for the ensemble bandit (cf. Figure 3). Here, we see that for both the groups, TS follows approximately the same trend as that of the optimal sampler, whereas UCB has a very random behavior. For group B, we see that the chances of each model getting selected are approximately equally likely, whereas for group A the likelihood of neighborhood arm being chosen is comparatively low.

**Figure 4:**
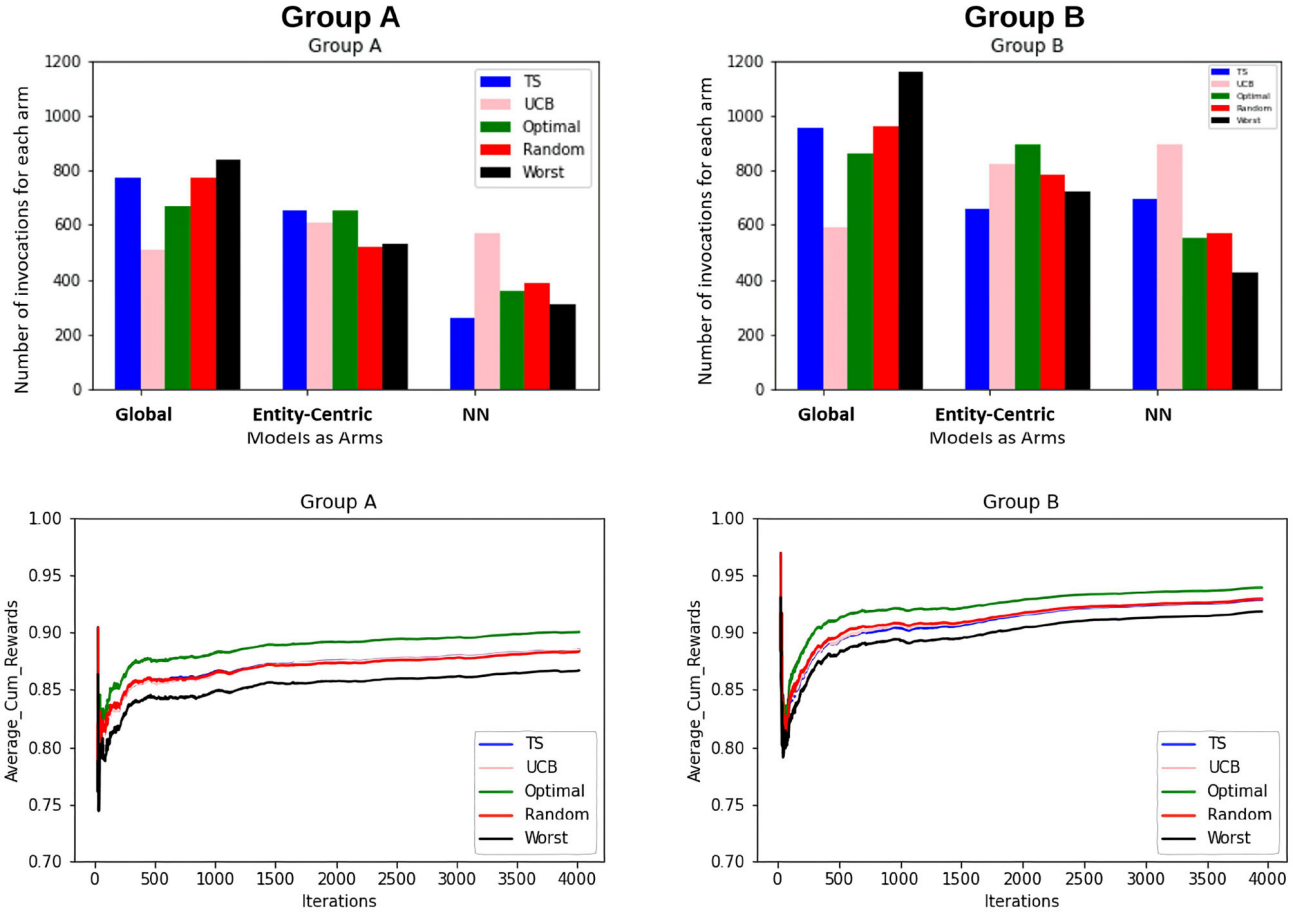
The figure shows the combined view of both the groups for the whole vector bandit w.r.t the number of times each arm is invoked for the items by different samplers and the average cumulative reward per iteration/time-step for those items. Five samplers as discussed in Section 3.3 above have been compared here.

Also, to be able to compare the whole vector bandit (cf. Figure 4) to that of an ensemble bandit, we are looking at the number of invocations for each type of arm (global, entity-centric, neighborhood) for both the configurations. Since the latter version has invocations for each item, we are calculating an average number of invocations for each arm to get an overview of its behavior. For that purpose, all the invocations were summed up and divided by the total number of items, i.e., 6 (cf. Table 1). Additionally, we are identifying the relative number of invocations for each item using a division by the sum of all the invocations which is 1680 and 2308 for groups A and B, respectively. We then can proceed to compare UCB in Table 3 for the ensemble bandit and the whole vector bandit. Similarly for, TS comparisons are made between the ensemble and the whole vector bandit in Table 4. We observe that the behavior of the bandits is very similar on average within the sampling strategy. On the other hand, when we compare UCB and TS we observe the preference of TS toward global arms whereas UCB prefers the local ones. To see which strategy is less optimal we compare the number of invocations to the optimal sampler (cf. Table 5) and see that TS has a behavior similar to that of the optimal one. This indicates the TS is more suitable for solving the problem since it aligns with the optimal strategy in all the configurations.

**Table 3:** Number of invocations for the global and local models by ensemble predictors and whole vector predictor using UCB as a sampling strategy.

		**Group A**				**Group B**			
		**Global**	**Entity**	**Neighborhood**	**Total**	**Global**	**Entity**	**Neighborhood**	**Total**
S02	*Absolute*	545	617	518	1680	480	863	965	2308
	*Relative*	32%	37%	31%	100%	21%	37%	42%	100%
S03	*Absolute*	511	627	542	1680	554	810	944	2308
	*Relative*	30%	37%	32%	100%	24%	35%	41%	100%
S04	*Absolute*	485	584	611	1680	601	822	885	2308
	*Relative*	29%	35%	36%	100%	26%	36%	38%	100%
S05	*Absolute*	512	569	599	1680	576	833	899	2308
	*Relative*	30%	34%	36%	100%	25%	36%	39%	100%
S06	*Absolute*	467	642	571	1680	661	847	800	2308
	*Relative*	28%	38%	34%	100%	29%	37%	35%	100%
S07	*Absolute*	531	590	559	1680	588	822	898	2308
	*Relative*	32%	35%	33%	100%	25%	36%	39%	100%
Total	*Absolute*	3051	3629	3400	-	3460	4997	5391	-
	*Average*	508.5	604.8	566.7	-	576.7	832.8	898.5	-
	*Relative*	30%	36%	34%	-	25%	36%	39%	-
Whole Vector	*Absolute*	506	607	567	1680	589	825	894	2308
	*Relative*	30%	36%	34%	100%	26%	36%	39%	100%

**Table 4:** Number of invocations for the global and local models by ensemble predictors and whole vector predictor using Thompson Sampling as a sampling strategy.

		**Group A**				**Group B**			
		**Global**	**Entity**	**Neighborhood**	**Total**	**Global**	**Entity**	**Neighborhood**	**Total**
S02	*Absolute*	802	494	384	1680	947	716	645	2308
	*Relative*	48%	29%	23%	100%	41%	31%	28%	100%
S03	*Absolute*	755	581	344	1680	972	671	665	2308
	*Relative*	45%	35%	20%	100%	42%	29%	29%	100%
S04	*Absolute*	756	544	380	1680	930	692	686	2308
	*Relative*	45%	32%	23%	100%	40%	30%	30%	100%
S05	*Absolute*	793	577	310	1680	962	706	640	2308
	*Relative*	47%	34%	18%	100%	42%	31%	28%	100%
S06	*Absolute*	790	517	373	1680	967	704	637	2308
	*Relative*	47%	31%	22%	100%	42%	31%	28%	100%
S07	*Absolute*	798	541	341	1680	987	677	644	2308
	*Relative*	48%	32%	20%	100%	43%	29%	28%	100%
Total	*Absolute*	4694	3254	2132	-	5765	4166	3917	-
	*Average*	782.3	542.3	355.3	-	960.8	694.3	652.8	-
	*Relative*	47%	32%	21%	-	42%	30%	28%	-
Whole Vector	*Absolute*	780	628	272	1680	970	649	689	2308
	*Relative*	46%	37%	16%	100%	42%	28%	30%	100%

**Table 5:** Number of invocations for the global and local models by ensemble predictors and whole vector predictor using optimal sampler as a sampling strategy.

		**Group A**				**Group B**			
		**Global**	**Entity**	**Neighborhood**	**Total**	**Global**	**Entity**	**Neighborhood**	**Total**
S02	*Absolute*	667	678	335	1680	862	895	551	2308
	*Relative*	40%	40%	20%	100%	37%	39%	24%	100%
S03	*Absolute*	675	627	378	1680	924	843	541	2308
	*Relative*	40%	37%	23%	100%	40%	37%	23%	100%
S04	*Absolute*	686	636	358	1680	914	910	484	2308
	*Relative*	41%	38%	21%	100%	40%	39%	21%	100%
S05	*Absolute*	714	611	355	1680	948	839	521	2308
	*Relative*	43%	36%	21%	100%	41%	36%	23%	100%
S06	*Absolute*	728	612	340	1680	887	893	528	2308
	*Relative*	43%	36%	20%	100%	38%	39%	23%	100%
S07	*Absolute*	747	582	351	1680	945	843	520	2308
	*Relative*	44%	35%	21%	100%	41%	37%	23%	100%
Total	*Absolute*	4217	3746	2117	-	5480	5223	3145	-
	*Average*	702.8	624.3	352.8	-	913.3	870.5	524.17	-
	*Relative*	42%	37%	21%	-	40%	38%	23%	-
Whole Vector	*Absolute*	670	653	357	1680	861	893	554	2308
	*Relative*	40%	39%	21%	100%	37%	39%	24%	100%

**Figure 5:**
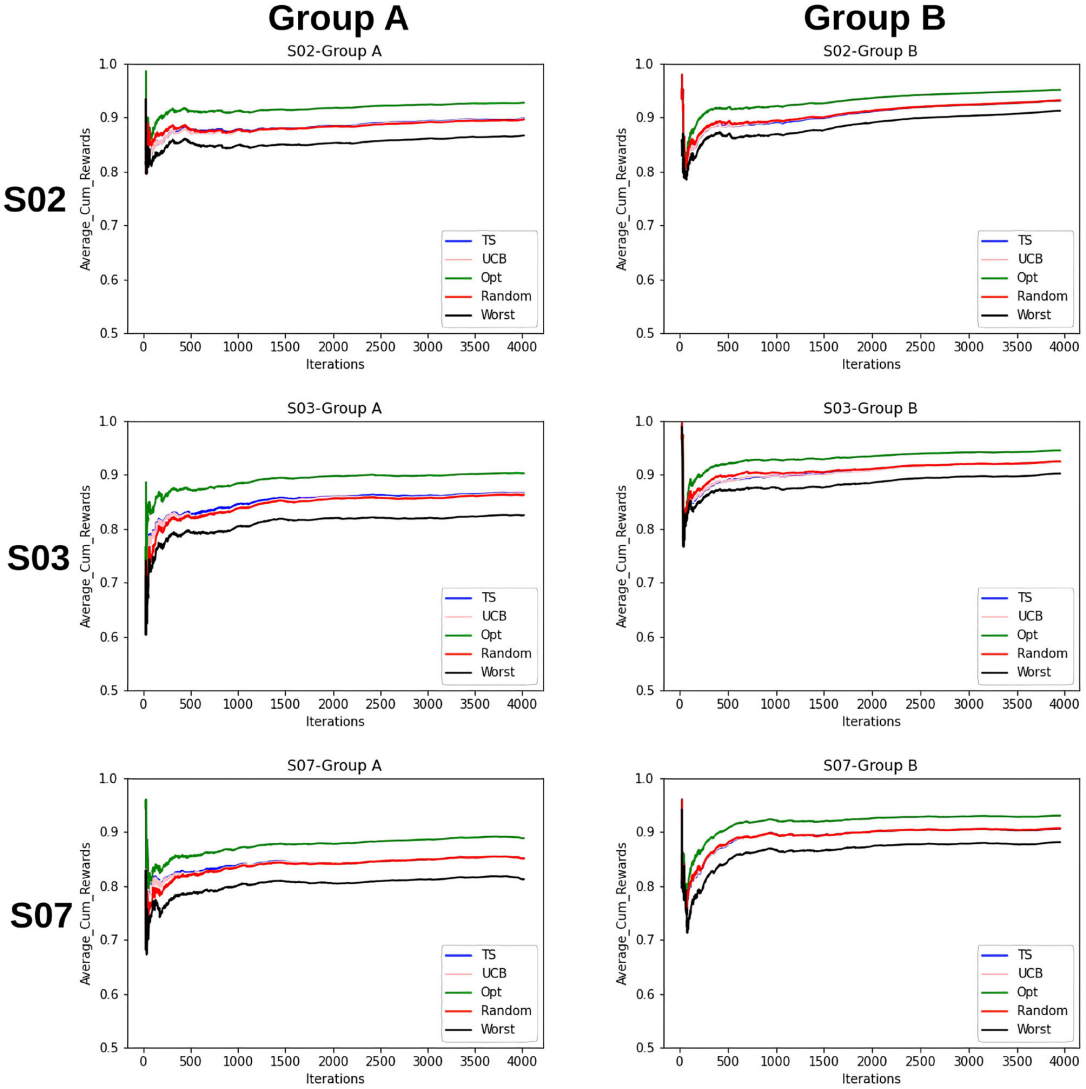
Average cumulative reward per iteration/time-step for the items for Group A and B. Here, the first column represents selections by Group A and the second column by Group B. Five samplers as discussed above have been compared here.

Furthermore, to better understand the convergence and therefore the learning behavior of the bandits, the average cumulative reward for each group is plotted. This provides an overview of the increase of rewards over time and therefore the increase in the quality of the models and the bandits' performance. This can be seen in Figure 5.

The findings that we derive from the average cumulative reward view of the Figure 5 are as follows:

1. For the random sampling strategy rewards increases with every iteration/time-step.
2. UCB and TS have a similar trend as that of a random strategy.
3. The investigated samplers are better than the worst samplers except at the very beginning where they do not have enough data to exploit.
4. Comparing the performances for group A and group B, we see that the lines for group B are closer to each other as compared to that for A. This indicates that the predictability for group B is slightly higher than that for group A.
5. This claim is supported by the fact that for group B on average cumulative rewards are higher than that for group A, which indicates fewer errors while predicting entities in group B.

**Figure 6:**
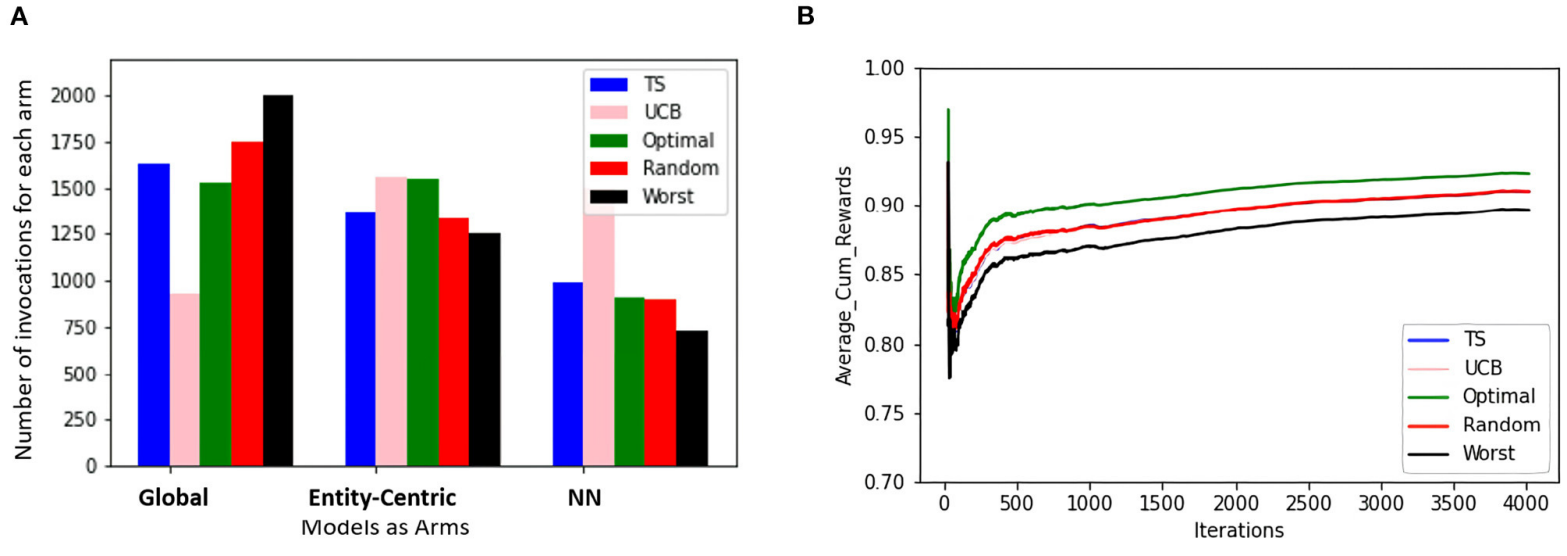
The figure shows the view for the simple bandit wherein the subfigure **(A)** shows the number of times each arm is invoked for all the items by different samplers for every entity. The subfigure **(B)** shows the average cumulative reward per iteration/time-step for all the entities. In both cases, 5 samplers have been compared wherein different colors represent different samplers.

**Figure 7:**
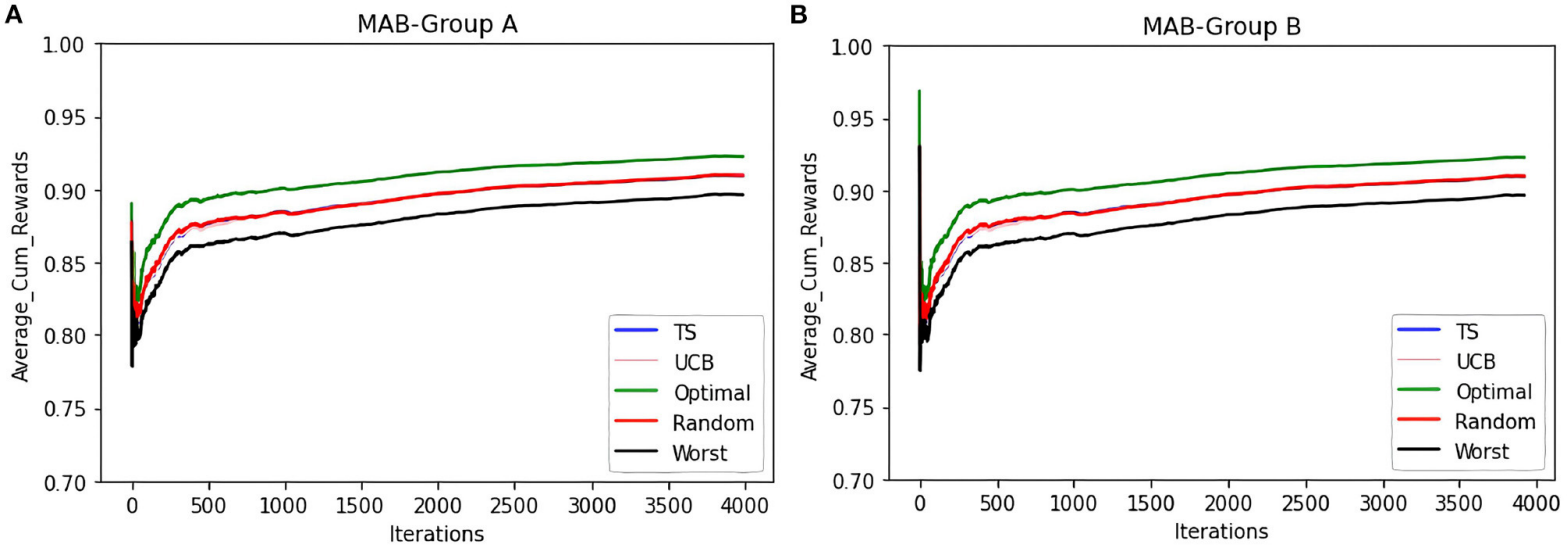
To see how the average cumulative reward in Figure 6B behaves for different groups, we split the behavior for both the groups which is shown in the subfigures **(A,B)**. *Y*-axis shows the average cumulative reward and the *X*-axis shows the time-steps and different colors represent 5 samplers that are compared here.

To compare the behavior of the bandit irrespective of the groups to which users belong, the same set of experiments are repeated without considering the group information. The same trends as that of the contextual multi-armed bandit are observed. Figure 6B shows that TS, UCB, and random samplers perform at par with each other. To see how this bandit behaves for the users in both groups A and B, the average cumulative rewards per iteration/time-step was plotted. We see in Figure 7 bandit learns well for both group A and group B users irrespective of the difference in the number of observations in both the groups.

In summary, it can be observed that local arms are preferred in many time-steps. There is no clear pattern in the choice between global and local arms but we found differences in the preference when using different sampling strategies. Additionally, the predictability of the groups varies slightly based on average cumulative rewards. To better understand how the bandit chooses between global and local arms, we investigate potential criteria in the next subsection.

### 4.3. Experiments for RQ 2

To be able to understand the behavior of the bandit, i.e., choice of the arms and development of the reward, we investigate different factors. These factors include the history length of the entities, their personal traits, their time of arrival in the system, and the temporal proximity of the predictions.

Initially, we are investigating the average error of each entity per item and prediction models underlying the arms. We observe in Table 6 that there is no clear boundary on the history length of the user where it can be clearly stated that local models are performing better on average than global models. Only entity 25 with a history length of 12 displays a clear preference to global models for all the items. Furthermore, also in Table 6 in the last column where we have the average error of all items per entity for different prediction models, this preference of entity 25 toward the global model can be confirmed. For entity 19 with a history length of 20, the global model performs on par with the neighborhood-based model. Beyond that, for most entities, the local models seem to perform better for all items except item S07, but their difference in performance to the global models is mostly negligible.

**Table 6:** Average error for each item across all the time steps using global, entity-centric, and neighborhood prediction models.

**Entities**	**Hist. length**	**S02**			**S03**			**S07**			**Total**		
		**Global**	**EC**	**NN**	**Global**	**EC**	**NN**	**Global**	**EC**	**NN**	**Global**	**EC**	**NN**
25	12	15.65	40.71	18.21	14.00	34.49	13.44	10.58	22.56	13.41	13.01	26.66	14.73
19	20	14.89	18.40	18.14	22.28	39.18	22.90	7.74	19.69	10.05	12.89	16.52	12.54
40	34	17.82	14.51	16.68	20.49	17.37	18.99	12.75	9.07	9.98	17.07	14.46	15.45
30	128	5.91	5.43	4.36	7.22	5.65	5.06	7.02	10.11	9.80	6.84	6.18	5.75
29	322	12.38	12.42	10.74	11.69	12.54	10.97	8.53	13.62	11.69	10.38	10.38	9.07
22	490	7.46	6.69	6.63	15.83	16.52	15.54	7.64	17.86	15.61	10.47	10.95	10.17

*For demonstration purposes, we include only items S02, S03, and S07*.

The behavior of S07 cannot be explained by the history length as a factor. Since the global model often performs better than the local models there seems to be a property that local models miss. One of these properties is the temporal proximity of the predictions. As can be seen in Table 1 the item S07 asks about how exhausted does the entity feel. This exhaustion might be dependent on external factors such as weather, time of day, etc., and, therefore, is more dependent upon the prior predictions rather than the entity itself.

**Figure 8:**
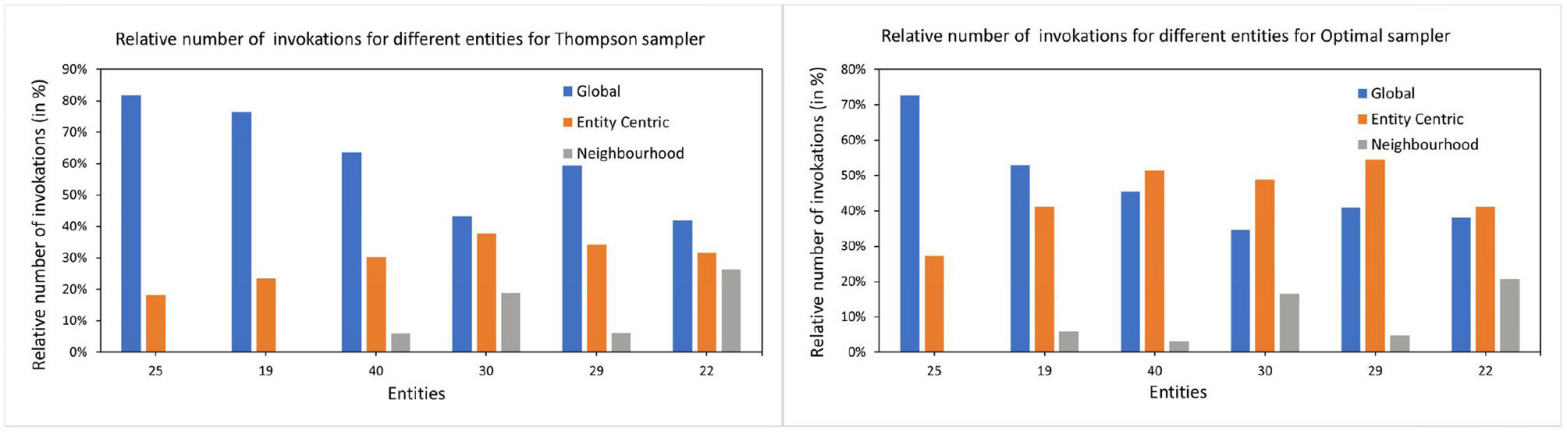
The relative number of invocations for global and local arms. The left subfigure shows the number of times each arm is invoked for all the items by TS samplers for selected entities. *Y*-axis shows the relative number of invocations and the *X*-axis are the entities. The right subfigure shows the same for the optimal sampler. For demonstration purposes, we have chosen 6 entities with varying history lengths.

The arrival time of the entity is interesting since the global model had more instances to predict already if the entity arrives later. Therefore, the global model might be already a better predictor for the late-arriving entities than the early birds. This cannot be proven since there seems to be no correlation between the arrival time of the entity and the preference toward the global model. This can be seen in Figure 8 where the choice of the arms is more dependent on the history length than the arrival time of the entity.

Since the analysis is only based on the individual arms of the bandit, we additionally checked the performance of the whole vector bandit to its arms. In Table 7, we compare the errors of optimal sampling strategy to that of TS and see that TS performs on average worse or equal to the best performing arm. This cannot be said about the optimal strategy though which specially for shorter history lengths perform better than its components. Additionally, for the longer history lengths, the error of the optimal strategy is close to the best arms. But for entity 22, which is also the first to arrive in the system, the optimal strategy is similarly better as for short history lengths. This might indicate that the initial predictions for entity 22 are better in a bandit setting but over time the performance of the individual arms averages out to be similar to that of an optimal sampler. Hence, the effect of the choice between the models is marginal at best, resulting in similar bandits and similar behavior as for a purely random model.

**Table 7:** Combined average error for each item across all the time steps for the complete bandit using optimal and Thompson sampling strategies to that of its arms.

**Entity-Id**	**Group**	**History length**	**Optimal**	**TS**	**Global**	**EC**	**NN**
25	B	12	11.72	13.75	13.01	26.66	14.73
19	A	20	11.21	12.87	12.89	16.52	12.54
40	A	34	13.22	15.59	17.07	14.46	15.45
30	A	128	5.45	6.61	6.84	6.18	5.75
29	B	322	8.68	10.18	10.38	10.38	9.07
22	A	490	8.91	10.32	10.47	10.95	10.17

*The users shown are chosen at random to have a mix of varying history lengths*.

Additionally, we investigated if there might be a cold start problem for the bandits, i.e., initializing the bandit with 0 rewards and invocations and, hence, having no information about the expected performance of each arm. To deal with this, the optimal strategy is used to kick start each of the samplers. Meaning for the prediction of the first 70 observations, the optimal strategy is employed to initialize UCB, random, and Thompson sampler with the resulting number of invocations and sum of rewards. The observed trend is similar to that in Figure 3. The same experiment is repeated by taking the first 140 observations instead of 70 for initialization purposes and still the trend is the same. This experiment further supports the claim made about similar behavior of UCB, TS, and random samplers.

In conclusion, we have shown that all 4 factors have influence on the behavior of the bandit and the quality of the predictions. The biggest influence has been on the history length and the temporal proximity of the predictions which will be discussed further in Section 5.

## 5. Discussion

### 5.1. Technical Perspective

As mentioned in Section 3.2, we incorporated local and global models as arms for CMAB which managed the invocations of those models to prove their effectiveness for RQ 1. A clear trend can be seen in Tables 3, 4, which prefers local models two third of the time for UCB and more than half for TS, whereas the latter is close in behavior to an optimal sampling strategy. The superiority of the local models is not necessarily large though. Only minor differences could be observed in Section 4.2. The sampling strategies including worst and optimal all are relatively close together in performance, meaning that the choice of models is not that significant. This might also be due to the late activation of local models as described in Section 3.2.1, since the fallback would be always the global model. A better strategy on when to chose each arm might still prove to be beneficial.

Additionally from Figures 3–5 we see that the context used in the CMAB does not make a big difference. Hence, the context of the items might be irrelevant to the problem or for the arms that were chosen. The sampling strategies might not be able to pick up relevant differences or models might be too close in performance. Furthermore, the predictability for group B is slightly higher than for group A for the CMAB. This might mean, that users of group B either formed a more distinct profile due to them exploring the app more, or their behavior is just simpler and therefore easier to predict. In both cases, the local arms might easier pick up the personal traits of each entity. Additionally, there is a difference in the usage of the context by the strategies. TS shows a significant difference in the choice of the neighborhood arm across groups. This implies TS can make use of homogeneity of group B. These findings are not significantly visible in the optimal sampler but indications can be found. The difference between the groups is not shared by the simple bandit as seen in Figure 7. Therefore, we can assume that context is important overall, but the given context is not sufficient, yet.

To understand which factors influence the performance between the models (RQ 2), we looked at the four parameters in Section 4.3. In Table 7, we found a clear dependency on the history length of the entities. This is on one hand due to the training time of the local models but also the fact that errors average out over time. Additionally, we checked if the arrival time of an entity in the system influences the choice of arms since the global arm will have a smaller error and higher reward at some point. Such a correlation does not exists, since Figure 8 only shows a dependency on the history length of the entity. The factors temporal proximity of the predictions and the personal traits of the entities might be in a trade-off between each other since local models might be able to better capture the personal traits but might not have recent predictions due to the behavior of the entities. In Table 6, most items for either local model perform better than the global one. We assume these items to be personal and therefore dependent on the traits of the entity. This seems to not be true for S07 where the global model oftentimes outperforms the local models. We explained in Section 4.3 that this might be due to external circumstances like weather and time. Due to that, context about the items might be beneficial for a CMAB.

As far as threats to validity are concerned, there is no guarantee that the multi-armed bandits converged to an optimal solution. This could be due to the lack of a fitting sampling strategy and the fact, that all models get better over time. For our experiments we assumed the data to follow the normal distribution and TS was designed accordingly. This assumption can be too stringent and hence we might just have a sub-optimal sampling strategy. Second, from a data perspective, there might be too few entities in the dataset, overall along with varying history lengths as entities with a small history might not be properly integrated by the bandit. This might have had a negative impact on the bandits and the models. This was further worsened due to the removal of entities without the group (context) information.

For future work, one main point should be therefore on the data collection process. Entities could be presented with the fact the recommendations from the mHealth app can be improved if they better work with the system, because for more active entities, their answers are easier to predict. Since the group as a context for the bandit did not provide useful insights, collecting additional meta-information about the lifestyle or similar information about the user might separate the groups inside the bandit better. Additionally, it might be helpful to get feedback from the entities on the aspect where the difference of the predictions to the actual answers are large. This could be directly incorporated into the reward function of the bandit and hence improving the sampling strategies. Given more time, the sampling strategies could have been enhanced by including information about the recent behavior of each user, because their differences might punish the rewards of yet not fully trained local models.

### 5.2. Mobile Health Perspective

From the perspective of optimal EMA prediction for personalized services, our findings demonstrate a clear trend: the optimal sampler of CMAB invokes the entity-centric model comparably often as the global model when it comes to predictions of the individual EMA through the ensemble (cf. Figure 3 and Table 5), while for the whole-vector prediction it invokes the entity-centric model equally or more often than the global one (cf. upper part of Figure 4). Our findings on how often the optimal sampler of the CMAB invokes each of the three models show a clear preference toward the entity-centric model. This means that predictions should be preferably based on the recordings of a user oneself, especially for users who (alike the participants of group B) deliver many recordings. Another formulation of this finding is that if an mHealth service provider has the option of invoking both a global model and an entity-centric one, then the latter should be preferred for users who interact intensively with the app—as soon as they start doing so.

One might argue that since the global model is used as often as the entity-centric model by the optimal sampler of the CMAB, then the two models are equally good. Unfortunately, this conclusion is not permitted. Rather, the global model is used so often, because many users contribute too few EMA, hence no personal, entity-centric models are available for them. As can be seen in the upper right subfigure of Figure 4, the optimal sampler invokes the entity-centric model is invoked more often than the global model for the users of group B, i.e., for the group the users of which contribute many EMA.

For the design of personalized services, as anticipated, e.g., in Vogel et al. (2021), this finding is rather disappointing, since it means that knowledge from other users is not very useful when it comes to prediction. This finding is also in contrast to the whole body of research on collaborative filtering for recommendation engines, where knowledge about similar users is exploited to predict a user's future preferences (Ricci et al., 2010), and to the advances on the potential of mHealth apps for decision support through prediction (Martínez-Pérez et al., 2014). However, our finding is less surprising when placed in the context of EMA: cutting-edge prediction technologies on temporal data build upon large amounts of recordings, see e.g. the sizes of the timestamped data sets used in Bellogín and Sánchez (2017); large data sets can be accumulated easily through sensor signals, as for the glycose predictor proposed by Pérez-Gandía et al. (2018), but are less easy to accumulate when users deliver EMA at their own discretion. Indeed, in our earlier work (Schleicher et al., 2020) on EMA recordings with the mHealth app TrackYourTinnitus (for short: TYT), we found that the majority of the users contributed less than three EMA in total.

Then, should we avoid learning from similar users for prediction? Since the neighborhood-based model is invoked comparatively rarely by the optimal sampler of the CMAB (cf. Table 5, Figure 3, and upper part of Figure 4), one is tempted to conclude that this model is inferior to the other two. However, the low number of invocations can also be explained by the limitations of the concrete study: the number of users is small in total, the period of observation is short, and the neighborhood-based model demands 5 observations per neighbor, in order to start making predictions. Hence, this model is available less often than the other two models. This means that if the population of users were larger and more EMA were available for some of them, then the aforementioned finding might be reversed. There are indeed indications in that direction: our earlier analyses on users of EMA-based mHealth apps for tinnitus (Unnikrishnan et al., 2019, 2020a, 2021) and diabetes (Unnikrishnan et al., 2020b) demonstrate that it is possible to exploit the data of users who deliver many EMA in order to do high-quality predictions for users who deliver few EMA (or are at the beginning of their interaction with the app). Nonetheless, choosing appropriate data to inform a neighborhood-based predictor is challenging (Unnikrishnan et al., 2021), not least because dependencies between past and current recordings do not generalize for the whole population of users. More research is needed to find ways of exploiting information on similar users for recommendations, when the available data are very sparse.

### 5.3. Experimentation Perspective and Insights for Tinnitus Research

Our investigation on the relative performance of global and local models is based on an experimental study. Hence, some of our findings are of relevance to the design of experiments involving mHealth app users, at least in the context of tinnitus research and of design of mHealth apps for tinnitus users.

First of all, there are differences in the predictability of different EMA items: for the prediction of EMA item S07, the optimal sampler selects the global model more often than the entity-centric one; for item S02, the entity-centric model is equally often selected for group A and more often selected for group B. This means that the global model, which is wholly insensitive to which user has contributed which EMA answer, delivers the best prediction (and is thus selected by the optimal sampler) as often as does the entity-centric model of that user.

Further, there are EMA items that were answered more often than others. More research is needed to shed light to the (un)popularity of some EMA items and to investigate whether some items can be consistently predicted from others; in our earlier work, we provide some indication to this end, by identifying Granger causalities among EMA items answered by TrackYourTinnitus users (Jamaludeen et al., 2021).

The findings on the answers to the EMA item S01 were remarkable. While the majority of users consistently answered that they perceived tinnitus every time they were asked, some users consistently answered that they do not, and some of the latter consistently answered that they do after they entered the second phase of the experiment. Since the number of users in this study was small, we do not attempt any conclusion from this finding. However, the presence of 4 out of 21 users with so different behavior with respect to this item shows that further research is needed, and in a larger pool of users, to better understand the role of this item.

The tips considered in the mHealth app TinnitusTipps were not personalized toward users, nor aligned to a user's previous answers to specific items. Hence, the effect of the tips on the answers to the EMA items cannot be assessed. However, group A was exposed to tipps from the beginning of the study, while group B was exposed only after the first two months. More analysis is needed to understand whether this can have resulted in the observed differences between group A and group B with respect to the number of contributed EMA recordings.

The optimal sampler of our CMAB demonstrated that the choice among models is influenced by the group (A vs. B). The difference between the two groups was that group B delivered more EMA recordings from the beginning on. Since the number of recordings influences the quality of the entity-centric predictor and of the neighborhood-based predictor, and (as a matter of fact) the quality of any multi-level model, it is advisable to quantify interaction intensity (cf. Schleicher et al., 2020) and to incorporate it into the learning model.

## Data Availability Statement

The data is part of the TinnitusTipps app developed by Sivantos, data can be shared upon request to the authors. Requests should be directed to WS: winfried.schlee@gmail.com.

## Ethics Statement

The studies involving human participants were reviewed and approved by Ethics Committee from the University Regensburg: 17-544-101. The patients/participants provided their written informed consent to participate in this study.

## Author Contributions

SS designed, implemented, and evaluated the technical components under the supervision of MS building upon and extending prior results of VU and MS created a conceptual model of the whole approach. VU contributed with ideas and insights from previous studies on the same data. RP, RH, and WS designed the mHealth app. RK and JS implemented it under the guidance of RP and provided data and instructions on data usage. WS lead the design of the two-armed mHealth study that resulted in the data set used in this investigation. RH delivered comments and feedback on the purpose of the app components and RP on the architecture and functionalities. SS and MS wrote the paper together. All other authors contributed with comments and feedback.

## Funding

This work was partially funded by the CHRODIS PLUS Joint Action, which has received funding from the European Union, in the framework of the Health Programme (2014-2020), Grant Agreement 761307 Implementing good practices for chronic diseases. This work was partially inspired by the European Union's Horizon 2020 Research and Innovation Programme, Grant Agreement 848261 Unification of treatments and Interventions for Tinnitus patients (UNITI). The development of the TinnitusTipps mHealth app was partially financed by Sivantos GmbH–WS Audiology. The funder was not involved in the study design, collection, analysis, interpretation of data, the writing of this article, or the decision to submit it for publication.

## Conflict of Interest

RH was employed by Sivantos GmbH–WS Audiology during the development of the mHealth app software. In this article, he provided comments and feedback on the description of the purpose of the app. The remaining authors declare that the research was conducted in the absence of any commercial or financial relationships that could be construed as a potential conflict of interest.

## Publisher's Note

All claims expressed in this article are solely those of the authors and do not necessarily represent those of their affiliated organizations, or those of the publisher, the editors and the reviewers. Any product that may be evaluated in this article, or claim that may be made by its manufacturer, is not guaranteed or endorsed by the publisher.

## Footnotes

^1^ https://tinnitustipps.lenoxug.de/
