# Supplementary material for: Predicting Ecological Momentary Assessments in an App for Tinnitus by Learning From Each User's Stream With a Contextual Multi-Armed Bandit
Source: Front Neurosci. 2022 Apr 11;16:836834. doi: 10.3389/fnins.2022.836834 (PMC9037687; doi:10.3389/fnins.2022.836834)
Supplement: Supplementary file 1 [file Data_Sheet_1.PDF]

Supplementary Material

1 SUPPLEMENTARY DATA

The material which could not be placed in the paper is discussed here. This mainly includes the meta data of the exploratory analysis and the details of the algorithms used.

The details of learning algorithms described in Section 3 of the main paper is as follows. For input instances $x_1, x_2, ..., x_n$, an incremental algorithm generates hypothesis $f_1, f_2, .., f_n$, so that f_{i+1} depends only on f_i and x_i. The first algorithm to be explored was a Hoeffding Tree (Hulten et al., 2001) which is an incremental, anytime decision tree induction algorithm. This algorithm works under the assumption that examples that are used to generate the distribution do not change over time. The main thing the trees make use of is that to choose an optimal splitting feature it is sufficient to have a small sample. Mathematically, this claim is backed up by the Hoeffding bound, which quantifies the number of instances that are required to approximate the statistics.

Table S1. Formula Explanation

Variables	Description
ϵ	Hoeffding bound
R	Range of a random variable. For a probability the range is 1, and for an information gain the range is log c, where c is the number of classes
δ	Confidence. 1 minus the desired probability of choosing the correct attribute at any given node
n	Number of samples

The formula for the Hoeffding bound is as in Eq. (S1) and the explanation of the same is in Table S1.

$$\epsilon = \sqrt{\frac{R^2 \ln(\frac{1}{\delta})}{2n}} \tag{S1}$$

The Hoeffding Trees guarantees the output which is similar to a non-incremental learner that takes into consideration infinite examples. The Hoeffding Regression Trees in order to decide the candidate for split take into consideration the computation of variance reduction. The homogeneity of the partitions is inversely proportional to the variance at the leaf nodes. Finally, either sample average or the preceptor (linear) is used as a predictor.

Hoeffding Adaptive Tree Regressor were also looked into. The only difference between normal Hoeffding Tree and the adaptive version is that for drift detection it uses ADWIN (Bifet and Gavaldà, 2007) which is an adaptive sliding window algorithm.

The third regressor that was explored was Adaptive Random Forest Regressor taking inspiration from Gomes et al (Gomes et al., 2017). The important points of the technique are 3 folds:

1. The re-sampling helps to give the heterogeneity
2. To split the nodes it also provides variety by selecting the subsets randomly
3. ADWIN to detect the drifts which allows the reset automatically. Also, it enables the alternate trees which are triggered when the warning is spotted and this alternate tree then replaces the current version as soon as warning turns into a drift

Table S2 provides the meta level information (median and the interquartile range) about the items with respect to both the groups.

Table S2. Median and interquartile ranges of the 6 items for the different groups

	**Group-A**		**Group-B**	
Item Id	Median	IQR	Median	IQR
S02	63	42	73	41
S03	33	38	58	47
S04	73	22	77	37
S05	28	29	26	39
S06	24	33	23	34
S07	39	29	25	34

2 SUPPLEMENTARY FIGURES

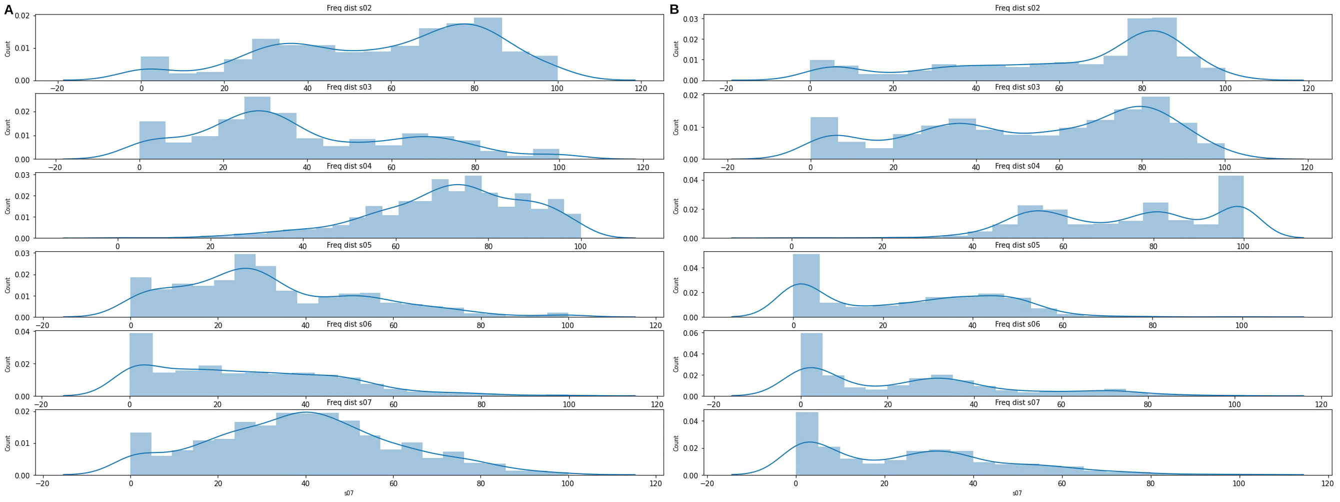

Figure S1. Comparison between distributions of group A and B for all the items (refers to the Uni-variate Analysis in the paper). **(A)** is Group A, **(B)** is Group B.

REFERENCES

Bifet, A. and Gavaldà, R. (2007). Learning from time-changing data with adaptive windowing. In *Proc. of the 2007 SIAM Int. Conf. on Data Mining (SDM'07)*. vol. 7, 443–448. doi:10.1137/1.9781611972771.42

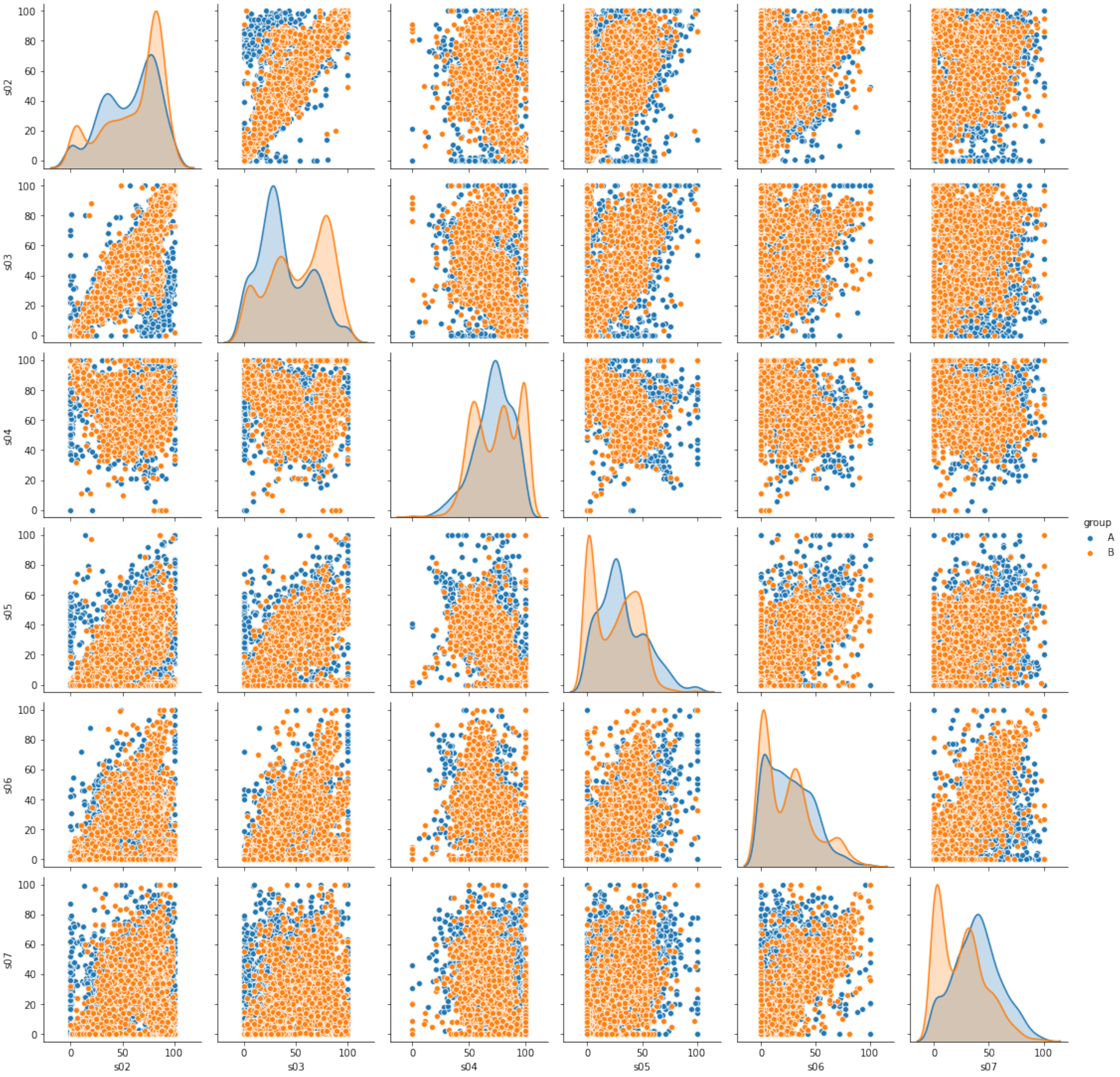

Figure S2. Figure shows the correlation between answers to EMA items w.r.t the groups. Here the main reason to choose a group is to see if the responses change in different groups. Blue dots/colors are used for Group A participants and orange for Group B.

Gomes, H. M., Bifet, A., Read, J., Barddal, J. P., Enembreck, F., Pfahringer, B., et al. (2017). Adaptive random forests for evolving data stream classification. *Machine Learning* 106, 1469–1495. doi:10.1007/s10994-017-5642-8

Hulten, G., Spencer, L., and Domingos, P. (2001). Mining time-changing data streams. In *Proc. of the 7th ACM SIGKDD Int. Conf. on Knowledge Discovery and Data Mining(KDD '01)* (ACM), 97–106. doi:10.1145/502512.502529
